# Nrf2-mediated mechanistic pathways of celastrol nephroprotection: disrupting the oxidative-inflammatory-apoptotic axis in cypermethrin toxicity

**DOI:** 10.3389/fphar.2025.1670444

**Published:** 2025-11-07

**Authors:** Ashraf Albrakati

**Affiliations:** Department of Human Anatomy, College of Medicine, Taif University, Ta’if, Saudi Arabia

**Keywords:** celastrol, oxidative, cypermethrin, apoptosis, antioxidant, inflammation

## Abstract

**Background:**

Cypermethrin, a widely used synthetic pyrethroid insecticide, accumulates in renal tissue causing kidney damage through incompletely understood mechanisms. This study evaluated celastrol’s nephroprotective effect against cypermethrin-induced kidney injury in rats.

**Methods:**

Five groups of male Wistar rats (n = 8 each) received daily treatments for 28 days: control, cypermethrin (25 mg/kg), celastrol (2 mg/kg), and celastrol + cypermethrin at low (1 mg/kg) or high (2 mg/kg) doses. Renal parameters, oxidative stress markers, inflammatory mediators, and apoptotic indicators were assessed using spectrophotometric assays, ELISA, qRT-PCR, and histology.

**Results:**

Cypermethrin impaired renal function, increased kidney weight, and elevated Kidney Injury Molecule-1 (KIM-1) levels. It significantly suppressed antioxidant defenses by reducing both the activities and mRNA expression of CAT, SOD, GPx, and GR, alongside GSH depletion and elevated oxidative markers (MDA, NO). Cypermethrin also downregulated the protein and gene expression of *Nfe2l2*, along with its downstream targets *Hmox1*, *GCLC*, and *NQO1*. Inflammatory responses were enhanced, as shown by upregulated TNF-α, IL-1β, NF-κB proteins and increased *NOS2* expression. Apoptosis was induced through elevated Bax, cytochrome c, and caspase-3 protein and gene expression, while both Bcl-2 protein and *Bcl2* mRNA were significantly reduced. Correlation analysis revealed significant inter-pathway connections, suggesting that oxidative stress as upstream trigger for inflammation and apoptosis. Celastrol treatment dose-dependently reversed these alterations, with the high dose restoring antioxidant and anti-apoptotic profiles more effectively than the low dose. Histopathological findings corroborated these results.

**Conclusion:**

Celastrol protects against cypermethrin nephrotoxicity through modulation of antioxidative, anti-inflammatory, and anti-apoptotic mechanisms. Correlation analysis suggests a potential role for Nrf2 in celastrol’s integrated nephroprotective effects.

## 1 Introduction

Nephrotoxicity represents a significant global health concern characterized by deteriorating renal function, resulting in metabolic waste accumulation, electrolyte imbalances, and increased risk of renal failure (Tang and Wei, 2024). Multiple factors contribute to kidney injury, including ischemia-reperfusion injury (Salvadori et al., 2015), nephrotoxic medications, sepsis (Malek and Nematbakhsh, 2015), and environmental toxicants (Yadav et al., 2024). Among environmental xenobiotics, cypermethrin, a widely used synthetic pyrethroid insecticide, has emerged as a concerning potential nephrotoxic agent with increasing clinical relevance (Abdou et al., 2012).

Cypermethrin is extensively used in agriculture for pest control in crops like cotton, vegetables, and cereals, as well as in public health programs for vector control (Farag et al., 2021). In many ecosystems, substances like cypermethrin accumulate through the food chain, ultimately affecting human health through chronic low-dose exposure (Chen et al., 2020). Tissue distribution studies have shown that cypermethrin concentrations are highest in the kidneys, followed by the liver, brain, and adipose tissue (Jabbar, 2022), raising significant concerns regarding kidney-specific toxicity during subchronic and chronic exposure periods (Farag et al., 2021).

The precise mechanisms of cypermethrin-induced nephrotoxicity remain incompletely understood. However, emerging evidence suggests oxidative stress plays a pivotal role in the pathogenesis of cypermethrin-induced renal injury (Afolabi et al., 2019). Prolonged exposure suppresses endogenous antioxidant defense systems (catalase (CAT), superoxide dismutase (SOD), glutathione peroxidase (GPx), and glutathione reductase (GR)) while enhancing reactive oxygen species (ROS) production. This persistent oxidative imbalance leads to lipid peroxidation, protein oxidation, and DNA damage, culminating in renal cellular dysfunction and death (Hussain et al., 2023).

Additionally, cypermethrin exposure activates inflammatory pathways, notably through nuclear factor-kappa B (NF-κB) signaling, resulting in pro-inflammatory cytokine production [tumor necrosis factor-α (TNF-α), interleukin-1β (IL-1β)] (Li et al., 2022). This inflammatory cascade exacerbates renal injury through progressive leukocyte infiltration and tubular damage. The mitochondrial apoptotic pathway, characterized by altered B-cell lymphoma 2 (Bcl-2)/Bcl-2-associated X protein (Bax) ratio, cytochrome c release, and caspase-3 activation, has been implicated in cypermethrin-induced renal cell death during continuous exposure (Wang et al., 2021).

Given widespread cypermethrin exposure and limited treatments for pesticide-induced nephrotoxicity, effective interventions are urgently needed.

Natural antioxidants have gained significant attention for mitigating nephrotoxicity by modulating oxidative stress, inflammation, and apoptotic signaling (Lee et al., 2024). Celastrol, a pentacyclic triterpene extracted from Tripterygium wilfordii, demonstrates potent antioxidant, anti-inflammatory, and anti-apoptotic properties (Song et al., 2023).

Celastrol’s protective effects stem from free radical scavenging and enhancement of endogenous antioxidant defenses through nuclear factor erythroid 2-related factor 2 (Nrf2) activation, while also regulating inflammatory mediators and inhibiting NF-κB pathways (Younis and Ghanim, 2022). Previous studies have shown celastrol has protective effects against liver injury (Sun et al., 2020), kidney injury (Younis and Ghanim, 2022), and other model diseases such as neurodegenerative disorders (Liu et al., 2022), but its potential against cypermethrin-induced nephrotoxicity and underlying molecular mechanisms remain largely unexplored.

This study aimed to investigate celastrol’s nephroprotective effects against cypermethrin-induced kidney injury in rats exposed for 4 weeks, hypothesizing that celastrol would attenuate renal damage by modulating oxidative stress, inflammatory responses, and apoptotic pathways during subacute exposure.

## 2 Materials and methods

### 2.1 Experimental animals

Forty male Wistar rats (weighing between 150 and 200 g) were obtained from the King Fahd Medical Research Center (KFMRC), King Abdulaziz University, Jeddah, Saudi Arabia. Prior to the experiment, animals were acclimatized for 1 week under standard laboratory conditions. Rats were housed in polypropylene cages at a temperature of 22 °C–25 °C with a 12-hour light/dark cycle and had free access to standard laboratory chow and water. Animals were monitored daily throughout the study for signs of toxicity including weight loss, or mortality.

#### 2.1.1 Animal grouping and treatment procedures

Animals were randomly divided into five groups (n = 8 per group). All treatments were administered orally via gavage once daily for 28 consecutive days. Cypermethrin and celastrol were purchased from Sigma-Aldrich Co. LLC, St. Louis, MO, United States.

The treatment groups were as follows:

Control group: Received vehicle (corn oil+2% DMSO) only as a vehicle control.

Celastrol (CEL) group: Rats received CEL (dissolved in corn oil at a dose of 2 mg/kg/day (Zhu et al., 2020).

Cypermethrin (CPM) group: Rats received CPM (dissolved in 2% DMSO) at a dose of 25 mg/kg/day, based on previous studies (Wang et al., 2019).

CEL-Low Dose + CPM group (CEL L D + CPM): Rats received CEL (1 mg/kg) 1 h prior to CPM (25 mg/kg) daily, starting from day 1.

CEL-High Dose + CPM (CEL H D + CPM): Rats received CEL (2 mg/kg) 1 h prior to CPM (25 mg/kg) daily, starting from day 1.

### 2.2 Anesthesia and sample collection

On day 29, all animals were anesthetized with ketamine (90 mg/kg) and xylazine (10 mg/kg) administered intraperitoneally. Kidneys were rapidly excised, rinsed with ice-cold saline, and processed for histological, biochemical, and molecular analyses.

### 2.3 Serum renal function tests

Blood samples were collected via cardiac puncture before sacrifice. Serum was separated by centrifugation at 3,000 rpm for 15 min. Renal function was assessed by measuring serum creatinine and blood urea nitrogen (BUN) using commercially kits (Randox Laboratories Ltd., Crumlin, County Antrim, United Kingdom). All assays were performed according to the manufacturers’ protocols.

### 2.4 Determination of kidney weight and relative kidney weight

The kidney weight was assessed based on the following mathematical calculation: (Almeer et al., 2019). Relative kidney weight= (Left kidney weight/Body weight) x 100.

### 2.5 Renal injury biomarkers

Kidney Injury Molecule-1 (KIM-1) was measured in kidney tissue homogenates using ELISA kit (Elabscience Biotechnology Inc., Houston, TX, United States) according to the manufacturer’s instructions.

### 2.6 Redox status determination

#### 2.6.1 Markers of oxidative imbalance

Oxidative stress markers were assessed in kidney tissue homogenates to evaluate the extent of cellular damage induced by cypermethrin exposure. Malondialdehyde (MDA) levels were determined using the procedure established by Ohkawa et al. (1979). Reduced glutathione (GSH) content was determined using the method outlined by Ellman (1959). Nitric oxide (NO) content in renal samples was measured by Griess reagent (Green et al., 1982).

#### 2.6.2 Markers of antioxidant response

To assess the antioxidant defense system, several key enzymatic activities were measured. SOD activity was evaluated using the technique described by Nishikimi et al. (1972). CAT activity was measured using the method of Lück (1965). GPx activity was assessed using the technique described by Paglia and Valentine (1967). Glutathione reductase (GR) activity was assessed using the technique described by Moron et al. (1979). Nuclear factor erythroid 2-related factor 2 (Nrf2) levels were quantified using ELISA kit (Elabscience Biotechnology Inc., Houston, TX, United States) according to the manufacturer’s instructions. Additionally, protein levels of heme oxygenase-1 (HO-1), glutamate-cysteine ligase catalytic subunit (GCLC), and NAD (P)H quinone oxidoreductase 1 (NQO1) were measured using respective ELISA kits.

### 2.7 Inflammatory markers

Inflammatory proteins in kidney tissue were quantified using commercial ELISA kits. TNF-α and IL-1β levels were measured with kits from BioVision Inc., Milpitas, CA, United States NF-κB protein levels were determined using an ELISA kit from Elabscience Biotechnology Inc., Houston, TX.

#### 2.7.1 Apoptotic markers

Apoptotic proteins in kidney tissue were quantified using commercial ELISA kits. Bcl-2 and Bax levels were measured with kits from BioVision Inc., Milpitas, CA, United States and expressed as ng/mg protein. Caspase-3 activity was determined with a colorimetric assay kit from BioVision Inc., Milpitas, CA, United States, while cytochrome c levels were assessed using an ELISA kit from Elabscience Biotechnology Inc., Houston, TX, United States to evaluate mitochondrial integrity. All assays were performed in accordance with the respective manufacturers’ protocols.

### 2.8 Gene expression analysis

Total RNA was extracted from kidney tissue samples using TRIzol reagent (Invitrogen, Carlsbad, CA, United States) according to the manufacturer’s instructions. RNA purity and concentration were determined spectrophotometrically using NanoDrop (Thermo Fisher Scientific, Waltham, MA, United States) by measuring absorbance at 260/280 nm. Complementary DNA (cDNA) was synthesized from 1 μg of total RNA using RevertAid™ H Minus Reverse Transcriptase following the manufacturer’s protocol. Quantitative real-time PCR (qRT-PCR) was performed using QuantiFast SYBR Green PCR kit (Qiagen, Hilden, Germany) on an Applied Biosystems 7,500 system (Thermo Fisher Scientific, CA, United States). The PCR cycling conditions included initial denaturation at 95 °C for 10 min, followed by 40 cycles of denaturation at 95 °C for 15s and annealing/extension at 60 °C for 60 s. All reactions were conducted in triplicate, and the relative gene expression levels were calculated using the 2^–ΔΔCt method with β-actin (Actb) as the internal control (Livak and Schmittgen, 2001). Target genes analyzed included antioxidant response markers (*Nfe2l2, Hmox-1, Sod2, Cat, Gpx-1, GSR, GCLC, NQO1*, inflammatory markers (*TNF-*α*, IL-1*β*, NF-κB*), nitrosative stress marker (*NOS2*), and apoptotic markers (Bax, Bcl-2, Caspase-3, Cytochrome c). Table 1 presents the primer sequences and accession numbers for the genes examined in this study.

**TABLE 1:** List of primer sequences of the genes analyzed by qRT-PCR.

Name	Accession number	Sense primer (5′→3′)	Antisense primer (5′→3′)
*Nfe2l2*	NM_031789.2	CAG​CAT​GAT​GGA​CTT​GGA​ATT​G	GCA​AGC​GAC​TCA​TGG​TCA​TC
*Hmox-1*	NM_012580.2	TTA​AGC​TGG​TGA​TGG​CCT​CC	GTG​GGG​CAT​AGA​CTG​GGT​TC
*Sod2*	NM_017051.3	AGC​TGC​ACC​ACA​GCA​AGC​AC	TCC​ACC​ACC​CTT​AGG​GCT​CA
*Cat*	NM_012520.2	TCC​GGG​ATC​TTT​TTA​ACG​CCA​TTG	TCG​AGC​ACG​GTA​GGG​ACA​GTT​CAC
*Gpx-1*	NM_030826.2	CGG​TTT​CCC​GTG​CAA​TCA​GT	ACA​CCG​GGG​ACC​AAA​TGA​TG
*NOS2*	NM_012611.3	GGT​GAG​GGG​ACT​GGA​CTT​TTA​G	TTG​TTG​GGC​TGG​GAA​TAG​CA
*TNF-α*	NM_013693.3	AGA​GGC​ACT​CCC​CCA​AAA​GA	CGA​TCA​CCC​CGA​AGT​TCA​GT
*GCLC*	NM_001498.4	TGG​AGG​ACC​GCT​ATG​AGG​AT	GCT​GGC​ATT​GTA​TTG​AGG​GA
*NQO1*	NM_017000.3	TGG​AGG​ACC​GCT​ATG​AGG​AT	GCT​GGC​ATT​GTA​TTG​AGG​GA
*IL-1β*	NM_008361.4	TGC​CAC​CTT​TTG​ACA​GTG​ATG	TTC​TTG​TGA​CCC​TGA​GCG​AC
*Bax*	NM_007527.3	CTGAGCTGACCTTGGAGC	GAC​TCC​AGC​CAC​AAA​GAT​G
*Bcl-2*	NM_009741.5	GAC​AGA​AGA​TCA​TGC​CGT​CC	GGT​ACC​AAT​GGC​ACT​TCA​AG
*Caspase-3*	NM_001,284,409.1	GAG​CTT​GGA​ACG​GTA​CGC​TA	CCG​TAC​CAG​AGC​GAG​ATG​AC
*Cytochrome c*	NM_012839.2	CTT​GGG​CTA​GAG​AGC​GGG​A	TGA​AGC​ACG​GGT​GAG​TCT​TC
*TLR-4*	NM_019178.2	TGG​ATA​CGT​TTC​CTT​ATA​AG	GAA​ATG​GAG​GCA​CCC​CTT​C
*β-Actin*	NM_007393.5	CTC​TAG​ACT​TCG​AGC​AGG​AGA​TGG	ATG​CCA​CAG​GAT​TCC​ATA​CCC​AAG​A
*NF-κB*	NM_009045.5	TGA​ACC​GAA​ACT​CTG​GCA​GCT​G	CAT​CAG​CTT​GCG​AAA​AGG​AGC​C
*GSR*	NM_000637.5	TAT​GTG​AGC​CGC​CTG​AAT​GCC​A	CAC​TGA​CCT​CTA​TTG​TGG​GCT​TG

### 2.9 Histopathological examination

Kidney samples were fixed in 10% neutral buffered formalin for 24 h, dehydrated through graded ethanol (70%, 80%, 90%, and 100% for 5 min each), cleared in xylene, embedded in paraffin wax, sectioned at 5 μm thickness, and stained with hematoxylin and eosin (H&E) (Drury and Wallington, 1980). The stained sections were evaluated under a light microscope (Nikon Eclipse E200) to assess pathological changes including glomerular alterations, tubular degeneration, inflammatory cell infiltration, and overall tissue architecture. Renal tissue sections underwent histopathological evaluation using a semi-quantitative scoring approach to assess structural alterations. The assessment employed the Endothelial–Glomerular–Tubular–Interstitial (EGTI) classification system as described by Toprak et al. (2020), which provides comprehensive evaluation of renal architectural components. Pathological changes were systematically graded using a standardized 5-point scale (0 = no changes, 1 = mild alterations, 2 = moderate damage, 3 = severe lesions, 4 = very severe disruption) for each anatomical compartment. Microscopic examination was conducted by an experienced pathologist in a blinded manner to eliminate assessment bias. Three randomly selected non-overlapping fields per tissue section were evaluated at ×400 magnification to ensure representative analysis. Histopathological scores were recorded and data presented as mean ± SEM.

### 2.10 Statistical analysis

All data are presented as mean ± SEM. Group comparisons were performed using one-way analysis of variance (ANOVA), followed by Tukey’s *post hoc* test to identify significant differences between treatment groups. Prior to correlation analysis, data normality was tested using the Shapiro-Wilk test. Spearman’s rank correlation coefficient (ρ) was used to evaluate the monotonic relationships between selected biochemical parameters. Correlation and all statistical analyses were conducted using SPSS software (version 26.0). A p-value less than 0.05 was considered statistically significant. All biochemical measurements were carried out in triplicates to ensure accuracy and reproducibility.

## 3 Results

### 3.1 Renal morphometric parameters

Morphometric analysis revealed significant alterations in kidney weight parameters across experimental groups. Cypermethrin administration significantly increased both absolute and relative kidney weights compared to controls (p < 0.05), indicating renal hypertrophy possibly attributed to cytotoxic effects or cellular edema. Celastrol treatment demonstrated dose-dependent nephroprotective effects, with high-dose co-administration normalizing kidney weight parameters to near-control levels, while low-dose treatment provided moderate but significant improvement compared to cypermethrin alone. Celastrol monotherapy showed no significant deviation from control values, suggesting its safety profile at therapeutic concentrations. These morphometric findings provide initial macroscopic evidence supporting celastrol’s nephroprotective properties against cypermethrin-induced renal toxicity (Table 2).

**TABLE 2:** Effects of CEL and CPM treatments on absolute and relative kidney weights in experimental groups.

Groups	Kidney weight (g)	Relative kidney weight
Control	0.85 ± 0.07	0.47 ± 0.02
CEL (2 mg/kg)	0.84 ± 0.06	0.46 ± 0.02
CPM (25 mg/kg)	1.2 ± 0.18^a^	0.67 ± 0.02^a^
CEL (1 mg/kg) + CPM (25 mg/kg)	1 ± 0.09^a^ ^,^ ^b^	0.56 ± 0.02^a^ ^,^ ^b^
CEL (2 mg/kg) + CPM (25 mg/kg)	0.91 ± 0.11^b^	0.50 ± 0.02^b^

Data are presented as mean ± SEM (n = 8).

^a^ Significantly different from control group (P < 0.05).

^b^ Significantly different from CPM, group (P < 0.05).

### 3.2 Renal function assessment

Biochemical assessment of renal function revealed significant alterations in key nephrotoxicity markers across experimental groups. Cypermethrin administration markedly elevated serum creatinine, blood urea nitrogen, and Kim-1 levels compared to controls (p < 0.05), indicating substantial renal dysfunction and tubular injury. Celastrol treatment demonstrated dose-dependent renoprotective effects, with high-dose co-administration significantly reducing all biomarker levels compared to cypermethrin alone, approaching near-normal values. Low-dose celastrol provided moderate but significant improvement in renal function parameters compared to cypermethrin monotherapy, though levels remained elevated relative to controls. Celastrol alone produced no significant changes in any renal function biomarkers, suggesting its nephrosafety profile. These biochemical findings collectively demonstrate celastrol’s capacity to mitigate cypermethrin-induced nephrotoxicity through preservation of glomerular filtration and tubular integrity (Table 3).

**TABLE 3:** Effects of CEL and CPM treatments on renal function biomarkers (Creatinine, BUN, and Kim-1) in experimental groups.

Groups	Creatinine (mg/dL)	Blood urea nitrogen (BUN) (mg/dL)	Kim-1 (ng/mL)
Control	0.89 ± 0.25	38 ± 5.81	22 ± 3.61
CEL (2 mg/kg)	0.87 ± 0.20	37.2 ± 4.10	21.4 ± 3.10
CPM (25 mg/kg)	1.80 ± 0.16^a^	68 ± 5.32^a^	80.31 ± 4.72^a^
CEL (1 mg/kg) + CPM (25 mg/kg)	1.29 ± 0.28^a^ ^,^ ^b^	50 ± 3.11^a^ ^,^ ^b^	38.0 ± 4.51^a^ ^,^ ^b^
CEL (2 mg/kg) + CPM (25 mg/kg)	1.02 ± 0.16^b^	40.9 ± 3.81^b^	29 ± 4.04^a^ ^,^ ^b^

Data are presented as mean ± SEM (n = 8).

^a^ Significantly different from control group (P < 0.05).

^b^ Significantly different from CPM, group (P < 0.05).

### 3.3 Oxidative stress markers and related gene expression

**FIGURE 1:**
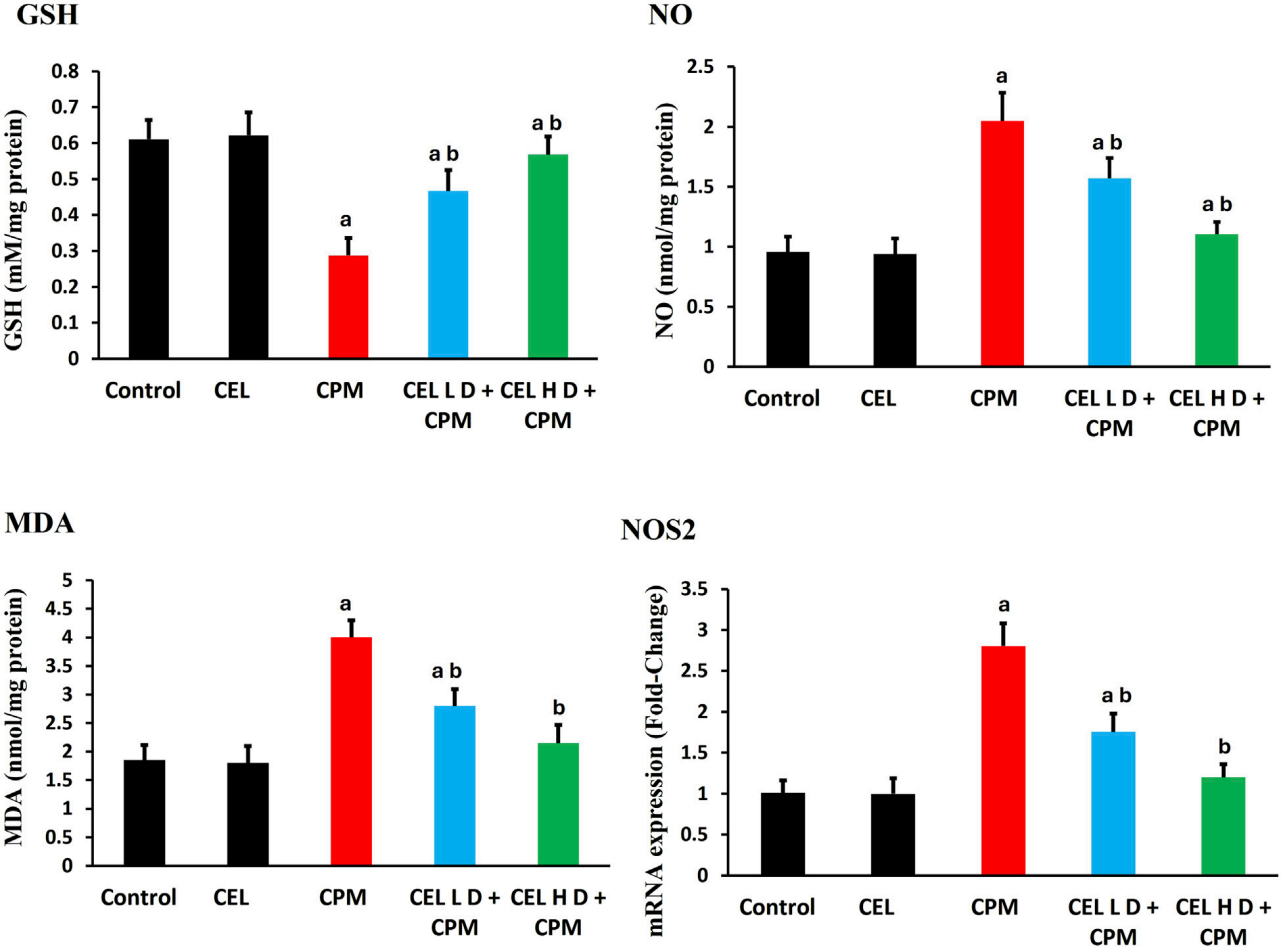
Effects of cypermethrin and celastrol on oxidative stress parameters in renal tissue. Rats were treated with cypermethrin and/or celastrol for 28 days. The figure shows levels of GSH, NO, MDA, and mRNA expression of inducible *NOS2*. Results are presented as mean ± SEM (n = 8). Statistical significance: ^a^ indicates significant difference compared to control (P < 0.05); ^b^ indicates significant difference compared to cypermethrin group (P < 0.05).

Cypermethrin exposure induced substantial redox disruption, with severe GSH depletion in CPM-treated animals (p < 0.05). Malondialdehyde levels exhibited marked elevation (p < 0.05), indicating extensive membrane damage. Nitric oxide levels increased significantly (p < 0.05), accompanied by *NOS2* mRNA upregulation (Figure 1).

Celastrol pre-treatment effectively mitigated these alterations. Both treatment protocols enhanced GSH concentrations and attenuated MDA accumulation (p < 0.05), with high-dose showing superior efficacy. NO levels and *NOS2* expression returned toward baseline in celastrol-treated groups (p < 0.05), with high-dose achieving near-complete normalization (Figure 1).

### 3.4 Antioxidant enzyme activities and corresponding gene expression profiles

**FIGURE 2:**
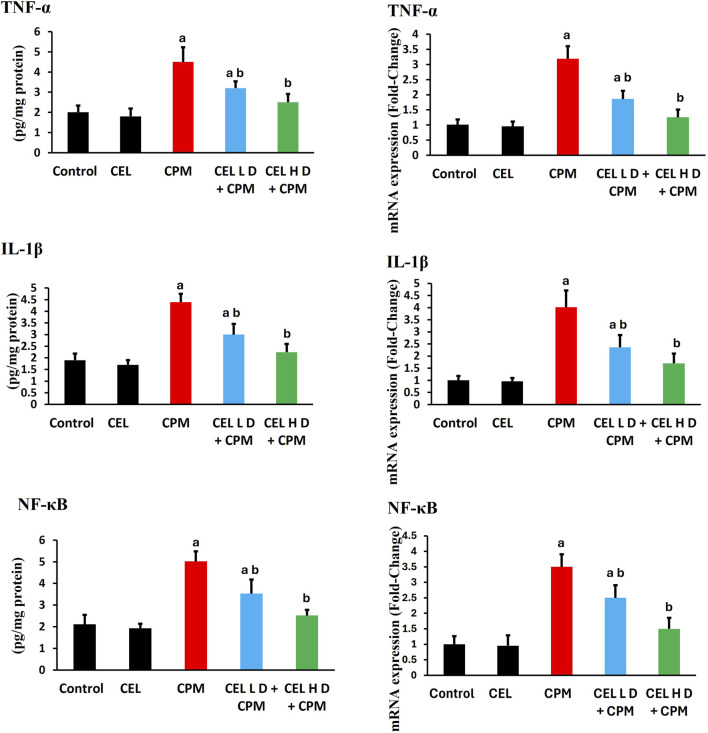
Effects of cypermethrin and celastrol on antioxidant enzyme activities and gene expression in renal tissue. Rats were treated with cypermethrin and/or celastrol for 28 days. The figure presents the enzymatic activities of CAT, GPx, SOD, and GR, along with the corresponding mRNA expression levels of *Cat*, *Gpx-1*, *Sod2*, and *GSR*. Results are shown as mean ± SEM (n = 8). Statistical significance: ^a^ indicates a significant difference compared to the control group (P < 0.05); ^b^ indicates a significant difference compared to the cypermethrin group (P < 0.05).

Analysis of antioxidant defense systems revealed significant dysregulation following cypermethrin exposure. Catalase protein levels demonstrated marked suppression in the CPM-treated group compared to control animals (p < 0.05), with corresponding downregulation of *Cat* gene expression (Figure 2). Glutathione peroxidase protein levels exhibited substantial reduction (p < 0.05), accompanied by parallel decrease in *Gpx-1* transcript abundance. Superoxide dismutase protein levels showed notable decline (p < 0.05), while *Sod2* gene expression displayed concomitant suppression. Additionally, glutathione reductase protein levels experienced significant inhibition relative to control values (p < 0.05), with associated reduction in *GSR* mRNA expression. These findings indicate coordinated transcriptional and translational suppression of key antioxidant enzymes (Figure 2).

Celastrol pre-treatment demonstrated remarkable protective efficacy against cypermethrin-induced alterations. Administration of celastrol at 2 mg/kg significantly elevated catalase protein levels compared to the cypermethrin-alone group (p < 0.05), with concurrent upregulation of *Cat* gene expression. Glutathione peroxidase protein levels showed substantial restoration following celastrol treatment (p < 0.05), accompanied by enhanced Gpx-1 transcript levels. Superoxide dismutase protein levels exhibited significant recovery (p < 0.05), while *Sod2* gene expression demonstrated parallel upregulation. Furthermore, glutathione reductase protein levels displayed marked improvement (p < 0.05), with corresponding enhancement of *GSR* mRNA abundance. These molecular and biochemical improvements collectively demonstrate celastrol’s capacity to restore antioxidant homeostasis through coordinated transcriptional activation and protein synthesis enhancement, with the higher dose protocol yielding superior therapeutic outcomes (Figure 2).

### 3.5 Nrf2 pathway regulation and downstream target expression

**FIGURE 3:**
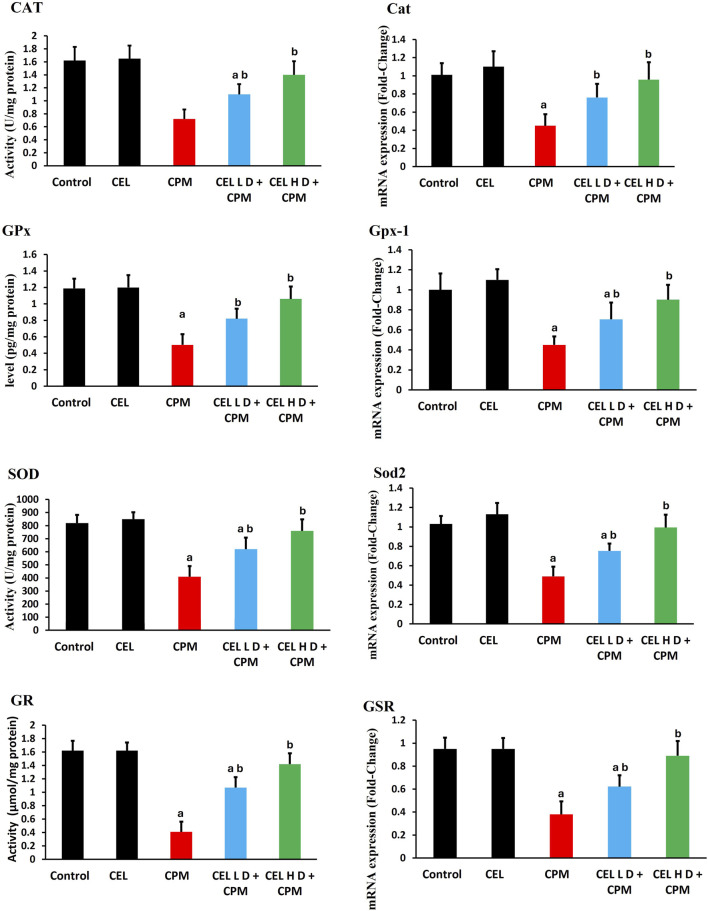
Effects of cypermethrin and celastrol on Nrf2 pathway and downstream target expression in renal tissue. Rats were treated with cypermethrin and/or celastrol for 28 days. The figure displays the protein levels of Nrf2, HO-1, GCLC, and NQO1, as well as the corresponding mRNA expression levels of *Nfe2l2*, *Hmox-1*, *GCLC*, and *NQO1*. Results are presented as mean ± SEM (n = 8). Statistical significance: ^a^ indicates a significant difference compared to the control group (P < 0.05); ^b^ indicates a significant difference compared to the cypermethrin group (P < 0.05).

Cypermethrin exposure severely disrupted Nrf2 antioxidant signaling. Nrf2 protein levels showed substantial reduction in CPM-treated animals (p < 0.05), with parallel suppression of *Nfe2l2* gene expression (Figure 3). HO-1 protein levels exhibited marked decline (p < 0.05), accompanied by corresponding downregulation of *H O -1* mRNA. *GCLC* protein levels demonstrated significant suppression (p < 0.05), while *GCLC* gene expression displayed concomitant reduction. Additionally, NQO1 protein levels experienced notable inhibition (p < 0.05), with associated decrease in *NQO1* transcript abundance.

Celastrol intervention effectively restored Nrf2 pathway functionality. Both protocols enhanced Nrf2 protein levels compared to CPM group (p < 0.05), with concurrent upregulation of *Nfe2l2* gene *expression*. HO-1 protein levels showed dose-dependent restoration (p < 0.05), accompanied by enhanced *H O -1* mRNA levels. GCLC protein levels exhibited significant recovery (p < 0.05), while *GCLC* gene expression demonstrated parallel improvement. Furthermore, NQO1 protein levels displayed marked enhancement (p < 0.05), with corresponding upregulation of *NQO1* transcript levels (Figure 3).

### 3.6 Inflammatory markers and related gene expression

**FIGURE 4:**
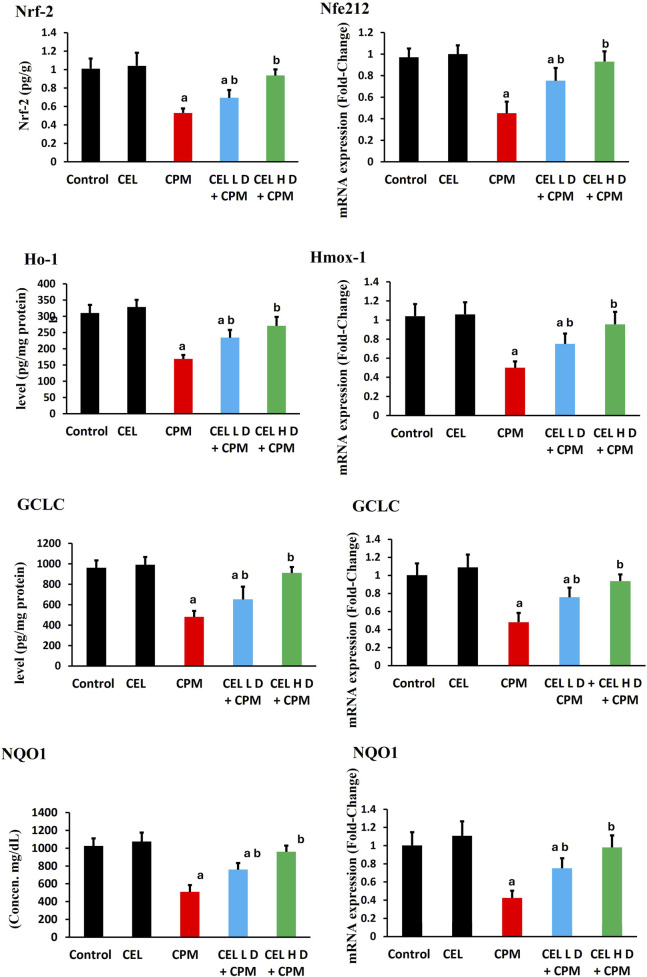
Effects of cypermethrin and celastrol on inflammatory markers in renal tissue. Rats were treated with cypermethrin and/or celastrol for 28 days. The figure presents the protein levels of TNF-α, IL-1β, and NF-κB, along with their corresponding mRNA expression levels. Results are shown as mean ± SEM (n = 8). Statistical significance: ^a^ indicates a significant difference compared to the control group (P < 0.05); ^b^ indicates a significant difference compared to the cypermethrin group (P < 0.05).

Cypermethrin induced robust inflammatory responses with marked elevation of pro-inflammatory mediators. TNF-α protein levels increased significantly in CPM-treated animals (p < 0.05), with concurrent upregulation of *TNF-*α gene expression showing greater fold-changes (Figure 4). IL-1β protein levels demonstrated substantial elevation (p < 0.05), accompanied by parallel enhancement of *IL-1*β mRNA levels. Additionally, NF-κB protein levels exhibited pronounced activation (p < 0.05), while *NF-κB* gene expression displayed corresponding upregulation, suggesting classical inflammatory signaling activation.

Celastrol pre-treatment demonstrated dose-dependent anti-inflammatory efficacy. Both regimens significantly reduced TNF-α protein levels compared to CPM group (p < 0.05), with concurrent suppression of *TNF-*α transcript levels. IL-1β protein levels showed marked attenuation following celastrol treatment (p < 0.05), accompanied by corresponding downregulation of *IL-1*β mRNA. Furthermore, NF-κB protein levels displayed substantial inhibition (p < 0.05), while *NF-κB* gene expression demonstrated parallel suppression. The dose-dependent attenuation indicates celastrol modulates inflammation through coordinated transcriptional and post-transcriptional mechanisms (Figure 4).

### 3.7 Apoptotic markers and related gene expression

**FIGURE 5:**
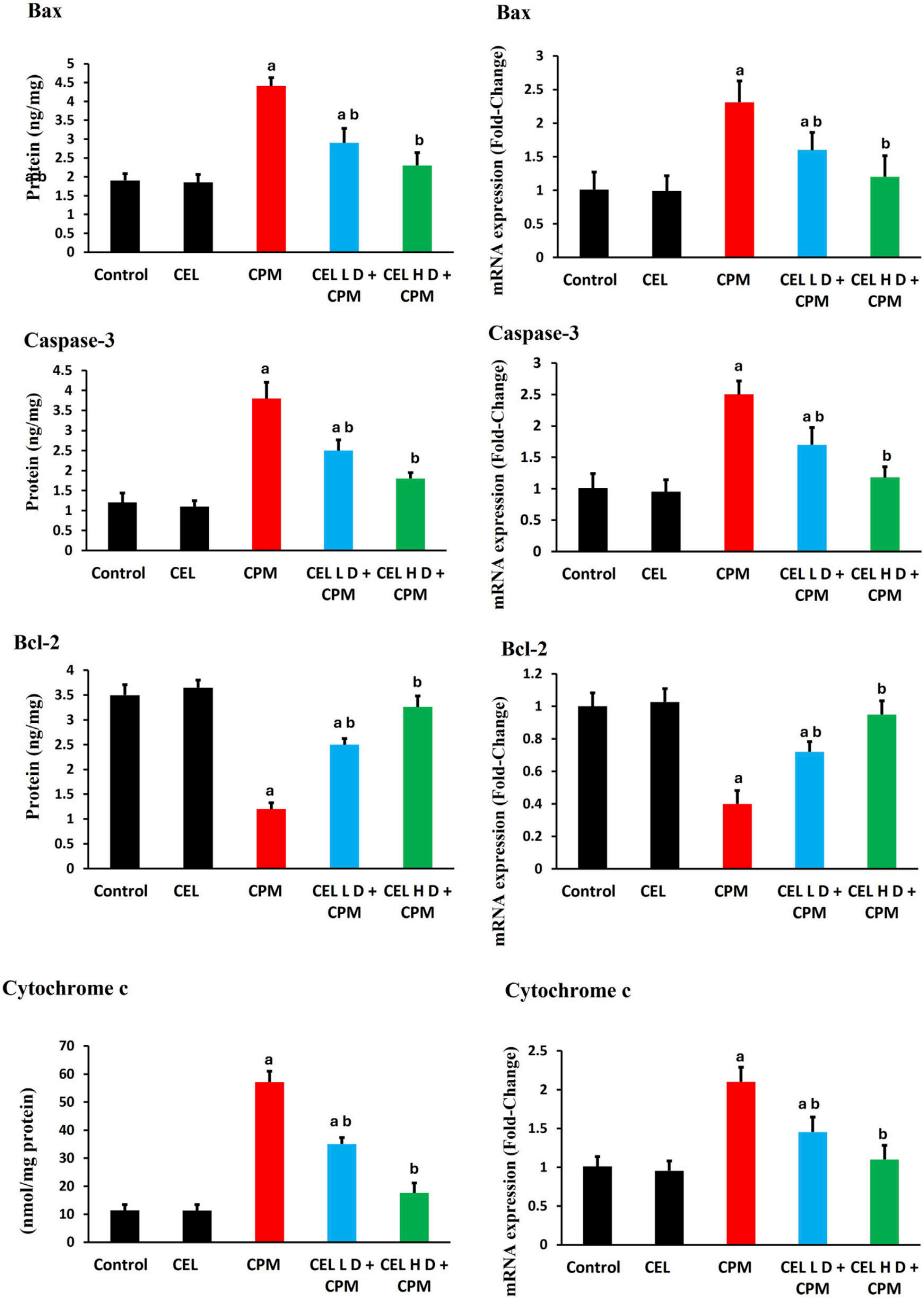
Effects of cypermethrin and celastrol on apoptotic markers in renal tissue. Rats were treated with cypermethrin and/or celastrol for 28 days. The figure presents the protein levels of Bax, caspase-3, Bcl-2, and cytochrome c, along with their corresponding mRNA expression levels. Results are shown as mean ± SEM (n = 8). Statistical significance: ^a^ indicates a significant difference compared to the control group (P < 0.05); ^b^ indicates a significant difference compared to the cypermethrin group (P < 0.05).

Cypermethrin triggered extensive apoptotic signaling in renal tissue. Bax protein levels showed substantial elevation in CPM-treated animals (p < 0.05), with concurrent upregulation of *Bax* gene expression. Bcl-2 protein levels demonstrated significant reduction (p < 0.05), accompanied by parallel suppression of *Bcl-2* mRNA levels, shifting the Bax/Bcl-2 ratio toward apoptosis. Caspase-3 protein levels exhibited pronounced activation (p < 0.05), while *caspase-3* gene expression displayed corresponding enhancement. Additionally, cytochrome c protein levels experienced dramatic elevation (p < 0.05), with associated upregulation of *cytochrome c* transcript levels, consistent with mitochondrial-mediated apoptosis (Figure 5).

Celastrol dose-dependently attenuated apoptotic signaling. Both protocols significantly reduced Bax protein levels compared to CPM group (p < 0.05), with concurrent downregulation of *Bax* gene expression. Bcl-2 protein levels showed marked restoration following celastrol treatment (p < 0.05), accompanied by enhanced *Bcl-2* mRNA levels. Furthermore, caspase-3 protein levels displayed substantial inhibition (p < 0.05), while *caspase-3* gene *expression* demonstrated parallel suppression. *Cytochrome c* protein levels exhibited significant reduction (p < 0.05), with corresponding downregulation of *cytochrome c* transcript abundance (Figure 5).

### 3.8 Correlation analysis between biochemical pathways

Correlation analysis revealed significant inter-pathway relationships suggesting the interconnected nature of cellular stress responses. The antioxidant regulator Nrf2 showed strong negative correlations with multiple biomarkers, indicating its potential protective role across various pathways. Specifically, Nrf2 exhibited strong negative correlations with oxidative stress markers including MDA (ρ = −0.82, p < 0.05) and nitric oxide levels (ρ = −0.72, p < 0.05), suggesting its possible role in combating oxidative damage.

The inflammatory pathway demonstrated significant inverse relationships with Nrf2, as evidenced by the strong negative correlation with NF-κB (ρ = −0.77, p < 0.05), indicating Nrf2’s anti-inflammatory protective effects. Similarly, apoptotic markers showed substantial negative correlations with the Nrf2 system, with caspase-3 displaying a correlation coefficient of ρ = −0.73 (p < 0.05), suggesting that enhanced Nrf2 activity provides significant protection against programmed cell death.

**FIGURE 6:**
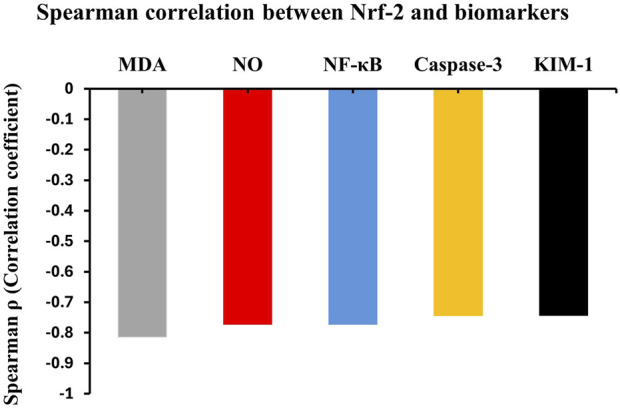
Inter-pathway correlation analysis in cypermethrin nephrotoxicity. Spearman’s correlation coefficients (ρ) between Nrf2 and key biochemical parameters. Nrf2 showed strong negative correlations with MDA (ρ = −0.82), NF-κB (ρ = −0.77), KIM-1 (ρ = −0.74), Caspase-3 (ρ = −0.73), and NO (ρ = −0.72). All correlations significant at p < 0.05. n = 8 per group.

The cellular proliferation marker KIM-1 also demonstrated a strong negative correlation with Nrf2 (ρ = −0.74, p < 0.05), indicating that Nrf2 activation may help maintain cellular integrity and prevent pathological proliferation. These consistent negative correlations across all measured biomarkers underscore the central protective role of Nrf2 as a master regulator of cellular defense mechanisms, coordinating antioxidant, anti-inflammatory, and anti-apoptotic responses in an integrated manner (Figure 6).

### 3.9 Histopathological examination

**FIGURE 7:**
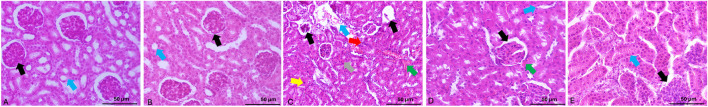
Microphotographs of H&E-stained renal tissue sections from the experimental groups. **(A,B)** Control and celastrol-only groups, respectively, demonstrate normal renal histology with intact glomeruli (black arrow) and normal tubules (blue arrow). **(C)** The cypermethrin group shows renal damage characterized by atrophy and cellular infiltration of the glomerular tuft (black arrows), congestion (gray arrow), leukocyte infiltration (green arrow), apoptosis (yellow arrow), tubular dilation (blue arrow), and necrotic areas (red arrow). **(D)** The CEL-Low Dose + CPM group shows reduced tubular damage (blue arrow), improved glomerular architecture (black arrow), and mild leukocyte infiltration (green arrow), reflecting partial protection. **(E)** The CEL-High Dose + CPM group exhibits essentially normal renal morphology with preserved glomeruli (black arrow) and normal tubules (blue arrow). H&E, scale bar = 50 μm.

Histopathological analysis revealed distinct morphological changes across groups (Figure 7). Control and celastrol-only groups displayed normal renal architecture with intact glomeruli and tubules, indicating celastrol’s safety (Figures 7A,B).

Cypermethrin-treated animals exhibited severe renal damage including glomerular atrophy with hemorrhage and congestion, cellular infiltration, marked leukocyte infiltration, tubular epithelial cloudy swelling, widespread apoptosis, and tubular dilation with hyaline casts (Figure 7C).

Celastrol pre-treatment provided dose-dependent protection. Low-dose (1 mg/kg + CPM) showed partial amelioration with reduced tubular injury and inflammatory infiltration, though some damage persisted (Figure 7E). High-dose (2 mg/kg + CPM) demonstrated near-complete preservation of renal morphology with minimal alterations and intact architecture (Figure 7D). These findings corroborate biochemical and molecular data, supporting celastrol’s dose-dependent nephroprotective efficacy.

The semi-quantitative histopathological analysis revealed that cypermethrin treatment significantly increased all renal damage parameters compared to the control group (p < 0.05), demonstrating moderate to severe scores for tubular dilatation, apoptotic cell abundance, glomerular atrophy, and leukocyte infiltration. Celastrol alone showed no significant histopathological changes compared to controls, indicating its safety profile at the tested dose.

Both celastrol pretreatment groups demonstrated significant protective effects against cypermethrin-induced nephrotoxicity in a dose-dependent manner. The low-dose celastrol group significantly reduced all damage parameters compared to the cypermethrin group (p < 0.05), though scores remained significantly elevated above control levels (p < 0.05). The high-dose celastrol group exhibited superior protective effects, with damage scores significantly lower than both the cypermethrin group and the low-dose celastrol group (p < 0.05), approaching near-normal levels comparable to controls, as demonstrated in Table 4.

**TABLE 4:** Semi-quantitative histopathological scoring of renal damage parameters in different experimental groups.

Group	Tubular dilatation	Apoptotic cell abundance	Glomerular atrophy	Leukocyte infiltration
Control	0.2 ± 0.42	0.2 ± 0.42	0.3 ± 0.67	0.2 ± 0.63
CEL (2 mg/kg)	0.2 ± 0.63	0.2 ± 0.63	0.3 ± 0.48	0.2 ± 0.42
CPM (25 mg/kg)	2.4 ± 1.30^a^ ^,^ ^b^	1.4 ± 1^a^ ^,^ ^b^	2.2 ± 1^a^ ^,^ ^b^	2.2 ± 0.1^a^ ^,^ ^b^
CEL (1 mg/kg) + CPM (25 mg/kg)	0.7 ± 0.82^a^ ^,^ ^b^	1 ± 0.81^a^ ^,^ ^b^	08 ± 0.63^a^ ^,^ ^b^	1.1 ± 0.87^a^ ^,^ ^b^
CEL (2 mg/kg) + CPM (25 mg/kg)	0.4 ± 0.48^a^ ^,^ ^b^	0.3 ± 0.67^a^ ^,^ ^b^	0.4 ± 0.69^a^ ^,^ ^b^	0.3 ± 0.67^a^ ^,^ ^b^

Lesions were graded on a 0–4 scale for each component (0 = none, 1 = mild, 2 = moderate, 3 = severe, 4 = very severe). Data presented as mean ± SEM (n = 8).

^a^ indicates significant difference from control group (p < 0.05).

^b^ indicates significant difference from cypermethrin group (p < 0.05).

## 4 Discussion

This study aimed to evaluate the nephrotoxic effects of cypermethrin and investigate celastrol’s protective role against cypermethrin-induced renal damage. Our findings revealed significant alterations at morphological, functional, biochemical, and molecular levels, and histopathological studies indicated cypermethrin’s nephrotoxicity and highlighted celastrol’s therapeutic potential.

The increased kidney weight observed after cypermethrin administration reflects inflammatory infiltration, cellular edema, and compensatory hypertrophy, consistent with Alibraheemi (2021) in pyrethroid toxicity models. Celastrol dose-dependently normalized these parameters, with high-dose treatment completely preventing renal hypertrophy. This morphometric protection aligns with celastrol’s anti-inflammatory properties documented by Yu et al. (2018), suggesting that reduced inflammatory infiltration preserves normal kidney architecture.

Cypermethrin exposure doubled serum creatinine and elevated BUN, indicating compromised glomerular filtration, consistent with Mossa et al. (2015). This preferential damage suggests cypermethrin accumulation during excretion causes direct cytotoxicity. Histopathological findings indicated that tubular damage precedes functional decline, consistent with Alalwani (2020). Celastrol dose-dependently attenuated these markers, with high-dose achieving near-normalization of KIM-1, demonstrating specific tubular protection through preservation of epithelial integrity.

Our findings provide substantial evidence that oxidative stress represents a central mechanism in cypermethrin-induced renal injury. The disruption of the antioxidant defense system was evident through suppression of enzymatic antioxidants, with CAT, GPx, SOD, and GR showing marked inhibition. This was accompanied by severe depletion of GSH content. Similar patterns have been documented by Taha et al. (2021), who demonstrated that 28-day sub-acute oral exposure of male albino rats to cypermethrin at 1/50 LD_50_ resulted in significant reductions in renal mitochondrial antioxidant enzyme activities (including GST and SOD) alongside increased lipid peroxidation and protein carbonylation, indicating systematic disruption of cellular redox homeostasis after pyrethroid exposure.

The consequences of this antioxidant defense collapse were manifested through elevation of oxidative damage markers, including MDA levels. Muthuviveganandavel et al. (2008) similarly reported that cypermethrin induces significant increases in lipid peroxidation markers. The membrane peroxidation may compromise renal epithelial cell integrity, potentially explaining the tubular damage observed, as suggested by Lieberthal and Levine (1996).

Concurrently, significant elevation in NO levels was observed following cypermethrin exposure, accompanied by marked upregulation of *NOS2* gene expression. This indicates coordinated dysregulation of nitrosative stress at both metabolic and genetic regulatory levels. The concurrent elevation of MDA and NO suggests synergistic oxidative and nitrosative damage.

Celastrol treatment dose-dependently normalized both NO production and *NOS2* expression, suggesting that modulation of nitrosative stress represents an important mechanism contributing to celastrol’s nephroprotective effects.

Celastrol intervention effectively restored redox balance through multiple complementary mechanisms. The dose-dependent normalization of antioxidant enzyme activities (CAT, GPx, SOD, GR) and GSH content suggests enhancement of endogenous antioxidant defense systems. Concurrently, the progressive reduction in oxidative damage markers (MDA and NO) indicates effective mitigation of free radical-mediated cellular injury. These findings align with reports by Sureshbabu et al. (2015) and Gao et al. (2024), who documented celastrol’s capacity to restore antioxidant defenses in various experimental models of oxidative stress-mediated tissue injury.

The molecular analysis revealed significant transcriptional dysregulation following cypermethrin exposure, providing mechanistic insight into the observed enzymatic impairments. The coordinated downregulation of multiple antioxidant genes, including Cat, Gpx-1, Sod2, and GSR, suggests suppression of a common transcriptional regulatory mechanism. Similar patterns of antioxidant gene suppression have been documented by Chong et al. (2016) in models of cypermethrin-induced oxidative stress.

The significant reduction in Nrf2 protein levels observed in our study provides mechanistic insight into the coordinated suppression of antioxidant genes. This protein reduction was accompanied by parallel downregulation of *Nfe2l2* gene expression, suggesting that cypermethrin disrupts this pathway at multiple regulatory levels. As the master regulator of cellular antioxidant defense, Nrf2 controls numerous cytoprotective genes through binding to antioxidant response elements. The suppression of downstream targets including *H O -1*, *GCLC*, and *NQO1* at both protein and mRNA levels indicates comprehensive pathway inhibition. This dysregulation of the Nrf2-ARE pathway appears central to the observed impairment of antioxidant defense systems, creating cellular vulnerability to oxidative damage, as reported by Chong et al. (2016).

Celastrol intervention effectively restored Nrf2 pathway functionality dose-dependently, with high-dose celastrol normalizing both Nrf2 protein levels and *Nfe2l2* expression. The parallel recovery of downstream targets (*H O -1, GCLC, NQO1*) suggests reactivation of the entire cytoprotective network. The relations between Nrf2 levels and oxidative stress markers highlight its central role in maintaining redox homeostasis. This Nrf2 activation likely represents a key mechanism behind celastrol’s nephroprotective effects, as similarly reported by (Ibrahim et al., 2024).

The correlation analysis provides compelling evidence for the interconnected nature of cypermethrin-induced nephrotoxicity mechanisms. The strong negative correlations between Nrf2 and NF-κB and caspase-3 validate Nrf2’s central role as a master protective regulator that coordinates defense against multiple cellular stress pathways. This finding supports the concept that Nrf2 dysfunction represents a critical upstream event that predisposes cells to both inflammatory activation and apoptotic cell death.

The inflammatory response to cypermethrin exposure was characterized by significant upregulation of pro-inflammatory mediators. TNF-α protein levels increased dramatically, with even greater fold-changes in mRNA expression, indicating transcriptional activation. Similar patterns were observed for IL-1β and the master regulator NF-κB. These findings align with Afolabi et al. (2019), who documented pyrethroid-induced inflammatory activation in renal tissue.

Celastrol intervention effectively suppressed this inflammatory response dose-dependently. The parallel normalization of both inflammatory and oxidative stress parameters suggests interconnected regulation of these pathways. The anti-inflammatory effects of celastrol, particularly through NF-κB suppression, have been well-documented by Zhang et al. (2019). Our observation that gene expression changes exceeded protein alterations suggests significant post-transcriptional regulation during toxic stress, with celastrol normalizing both processes. These findings are consistent with reports by Shrivastava et al. (2015), who documented celastrol’s capacity to modulate apoptotic signaling.

Our findings establish the mitochondrial apoptotic pathway as a significant mechanism in cypermethrin-induced nephrotoxicity. The observed upregulation of pro-apoptotic Bax coupled with downregulation of anti-apoptotic Bcl-2 indicates disruption of the critical Bax/Bcl-2 balance. This imbalance was associated with significant cytochrome c release and caspase-3 activation, reflecting coordinated apoptotic activation. Correlation analysis revealed that Nrf2 showed strong negative correlation with the apoptotic executor caspase-3, indicating that suppressed antioxidant defenses contribute to apoptotic activation. Similar relationships between oxidative stress, inflammation, and apoptosis in pyrethroid toxicity have been documented by Li et al. (2023).

Celastrol treatment effectively mitigated apoptotic changes dose-dependently, normalizing the Bax/Bcl-2 ratio, reducing cytochrome c release, and suppressing caspase-3 activation. This anti-apoptotic effect appears mediated through Nrf2 pathway restoration, as demonstrated by the strong negative correlation between Nrf2 and apoptotic markers. The coordinated protection involves enhanced antioxidant defenses and reduced inflammation, as evidenced by Nrf2’s negative correlations with oxidative stress markers (MDA) and inflammatory mediators (NF-κB). These findings are consistent with reports by Shrivastava et al. (2015), who documented celastrol’s capacity to modulate apoptotic signaling.

The histopathological findings validate our biochemical results. Severe renal damage in cypermethrin-treated animals—including glomerular atrophy, tubular dilation, and inflammatory infiltration—directly correlates with elevated KIM-1 and impaired function, aligning with Mamun et al. (2014) who reported similar changes in pyrethroid nephrotoxicity. Celastrol’s dose-dependent histological protection paralleled biochemical improvements, with high-dose achieving near-complete architectural preservation. This protective effect is comparable to Wu et al. (2018), who demonstrated celastrol prevented LPS-induced tubular damage, and Nie et al. (2020), who showed celastrol preserved glomerular structure in diabetic nephropathy.

These correlation patterns validate our mechanistic model and provide statistical evidence for the coordinated therapeutic approach demonstrated by celastrol’s multi-pathway protective effects. Collectively, correlation analysis revealed strong negative relationships between Nrf2 and all damage markers: MDA, NF-κB, KIM-1, caspase-3, and NO. These consistent correlations underscore Nrf2’s central protective role and validate celastrol’s multi-pathway therapeutic mechanism.

Comparative analysis between celastrol doses reveals important therapeutic insights. The higher dose demonstrated superior efficacy, achieving near-complete normalization of renal function markers (creatinine) and better restoration of antioxidant enzymes. However, modest differences between doses suggest a narrow therapeutic window, with celastrol’s nephroprotective effects potentially plateauing around 2 mg/kg, indicating optimal dosing rather than dose escalation should be the therapeutic focus.

A limitation of the present study is that functional validation of Nrf2 signaling was not performed through genetic or pharmacological inhibition. Thus, while our findings strongly suggest an association between Nrf2 activation and celastrol’s nephroprotective effects, they remain correlative. Future studies using Nrf2 knockdown or inhibitors will be required to establish direct causality.

## 5 Conclusion

This study indicates that sub-acute cypermethrin exposure induces nephrotoxicity through three interconnected pathways: suppression of Nrf2-mediated antioxidant defenses with oxidative/nitrosative stress induction, NF-κB inflammatory cascade activation, and mitochondrial-mediated apoptosis. Correlation analysis suggests that oxidative stress may serve as an upstream trigger for inflammation and apoptosis, while Nrf2 potentially exerts protective effects across cellular pathways, culminating in tubular damage and renal dysfunction. Celastrol emerged as a promising nephroprotective agent through apparent modulation of multiple pathways, including association with Nrf2 signaling, enhancing antioxidant defenses, suppressing inflammation, and inhibiting apoptosis. These observations support celastrol’s integrated therapeutic approach for mitigating pyrethroid nephrotoxicity, though functional validation studies are needed to establish these mechanisms and indicate its translational promise for preventing pesticide-induced renal injury.

## Data Availability

The original contributions presented in the study are included in the article/supplementary material, further inquiries can be directed to the corresponding author.
